# Association of the combined triglyceride glucose-body mass index and serum IQGAP3 in appraising coronary lesion severity in type 2 diabetes

**DOI:** 10.3389/fendo.2026.1898396

**Published:** 2026-07-02

**Authors:** YuRong Sun, Jingsi Zhang, Yi Lu, Yeting Chang, Yanchun Ding

**Affiliations:** Department of Cardiology, The Second Hospital of DaLian Medical University, Dalian, Liaoning, China

**Keywords:** coronary artery disease, insulin resistance, IQGAP3, TyG-BMI, type 2 diabetes mellitus

## Abstract

**Background:**

IQGAP3 is a novel scaffold protein involved in inflammatory and metabolic regulation and may contribute to atherosclerosis. However, its clinical significance in coronary artery disease among patients with type 2 diabetes mellitus (T2DM) remains unclear. This study investigated the association between serum IQGAP3 and coronary lesion severity and evaluated the diagnostic value of combining IQGAP3 with the triglyceride glucose-body mass index (TyG-BMI).

**Methods:**

This prospective study enrolled T2DM patients undergoing coronary angiography. Serum IQGAP3 levels were measured by enzyme-linked immunosorbent assay, and TyG-BMI was calculated using routine clinical parameters. Coronary lesion severity was assessed using the Gensini score and categorized by tertiles. Multivariable logistic regression, restricted cubic spline (RCS), subgroup, and interaction analyses were performed. ROC curves, net reclassification improvement (NRI), and integrated discrimination improvement (IDI) were used to assess model performance.

**Results:**

A total of 392 patients were included. Serum IQGAP3 levels and TyG-BMI increased significantly across Gensini score tertiles (all P< 0.05). Both IQGAP3 (OR = 3.119, 95% CI: 2.049–4.747, P< 0.001) and TyG-BMI (OR = 1.012, 95% CI: 1.003–1.021, P = 0.006) were independently associated with severe coronary stenosis. Adding TyG-BMI to the traditional risk factor model improved discrimination (AUC: 0.702 vs. 0.753; NRI = 0.180, IDI = 0.065; both P< 0.001). Further incorporation of IQGAP3 increased the AUC to 0.803 and significantly improved reclassification (NRI = 0.307, IDI = 0.138; both P< 0.001), with optimism-corrected internal validation demonstrating an AUC of 0.788 and a calibration slope of 0.926. RCS analyses demonstrated a linear positive association between IQGAP3 and disease risk, whereas TyG-BMI exhibited a nonlinear relationship. Subgroup analyses showed consistent associations across most clinical strata. Age significantly modified the association between IQGAP3 and severe coronary stenosis (Pinteraction = 0.036), with a stronger effect observed in patients younger than 65 years.

**Conclusion:**

Serum IQGAP3 and TyG-BMI are independently associated with coronary lesion severity in T2DM patients. Their combined assessment provides incremental discriminative value for coronary lesion severity and may serve as a practical, non-invasive tool for cardiovascular risk stratification.

## 1 Introduction

Type 2 diabetes mellitus (T2DM) is a complex metabolic disorder characterized by chronic hyperglycemia and insulin resistance, which trigger systemic micro- and macrovascular complications. The resulting high morbidity and mortality, particularly from coronary heart disease (CHD), impose a substantial global clinical and economic burden (1, 2). In patients with concurrent T2DM and CHD, persistent glucometabolic derangements disrupt the vascular endothelial barrier and activate inflammatory cascades. This cytokine-mediated inflammatory response directly drives and accelerates atherosclerotic plaque progression (3). Consequently, early and precise assessment of coronary atherosclerotic burden is vital for improving clinical risk stratification and tailoring therapeutic strategies in this high-risk population. Although coronary angiography combined with the Gensini score remains the benchmark for quantifying luminal stenosis and plaque burden, its routine use in screening and longitudinal surveillance is constrained by its invasiveness and the risk of contrast-induced injury (4, 5). This clinical limitation underscores the urgent need for novel, non-invasive, cost-effective, and sensitive peripheral blood biomarkers to predict coronary artery disease evolution in T2DM patients.Insulin resistance (IR) is a foundational driver of T2DM and metabolic dysregulation, closely linked to the initiation and progression of coronary atherosclerosis (AS) (6). While the hyperinsulinemic-euglycemic clamp remains the diagnostic gold standard for IR, its technical complexity and time-consuming nature preclude routine clinical use or large-scale screening (7). Consequently, the triglyceride glucose-body mass index (TyG-BMI) has emerged as a pragmatic alternative, capturing complementary pathways of systemic metabolic load and obesity while demonstrating strong correlations with cardiovascular risk factors like hypertension (8, 9). However, because atherosclerotic evolution in T2DM involves intricate, localized inflammatory networks—including endothelial injury, lipid accumulation, and vascular smooth muscle cell (VSMC) proliferation (10, 11)—a solitary metabolic index possesses inherent prognostic blind spots. Synergizing TyG-BMI with complementary biomarkers is thus essential to refine the precision and comprehensiveness of coronary severity assessments in this population.Enhancing this predictive efficacy requires capturing early signals of structural vascular remodeling. Beyond its documented role in oncogenesis (12), IQ motif-containing GTPase-activating protein 3 (IQGAP3)—a scaffold protein modulating cytoskeleton dynamics, proliferation, and migration—is gaining traction in cardiovascular biology, emerging evidence indicates that the interaction between the Kv1.3 channel and IQGAP3 bridges membrane electrical signaling and VSMC phenotypic remodeling; specifically, depolarization-driven Kv1.3-IQGAP3 coupling provides the molecular impetus for VSMC dedifferentiation and hyperproliferation (13). Mechanistically, pairing TyG-BMI (reflecting systemic metabolic burden) with serum IQGAP3 (marking downstream vascular wall remodeling and proliferative status) may offer a synergistic paradigm to optimize the prediction of coronary lesion evolution in patients with T2DM.

While serum IQGAP3 and the TyG-BMI index are recognized markers of vascular and metabolic stress, their combined clinical utility in patients with concurrent T2DM and CHD remains uncharacterized. Utilizing a cohort of T2DM patients undergoing coronary angiography, this study is the first to investigate the association of peripheral serum IQGAP3 levels and the TyG-BMI index with the severity of coronary stenosis. The findings aim to provide novel evidence-based insights for early risk stratification in this high-risk population.

## 2 Methods

### 2.1 Study population

**Figure 1:**
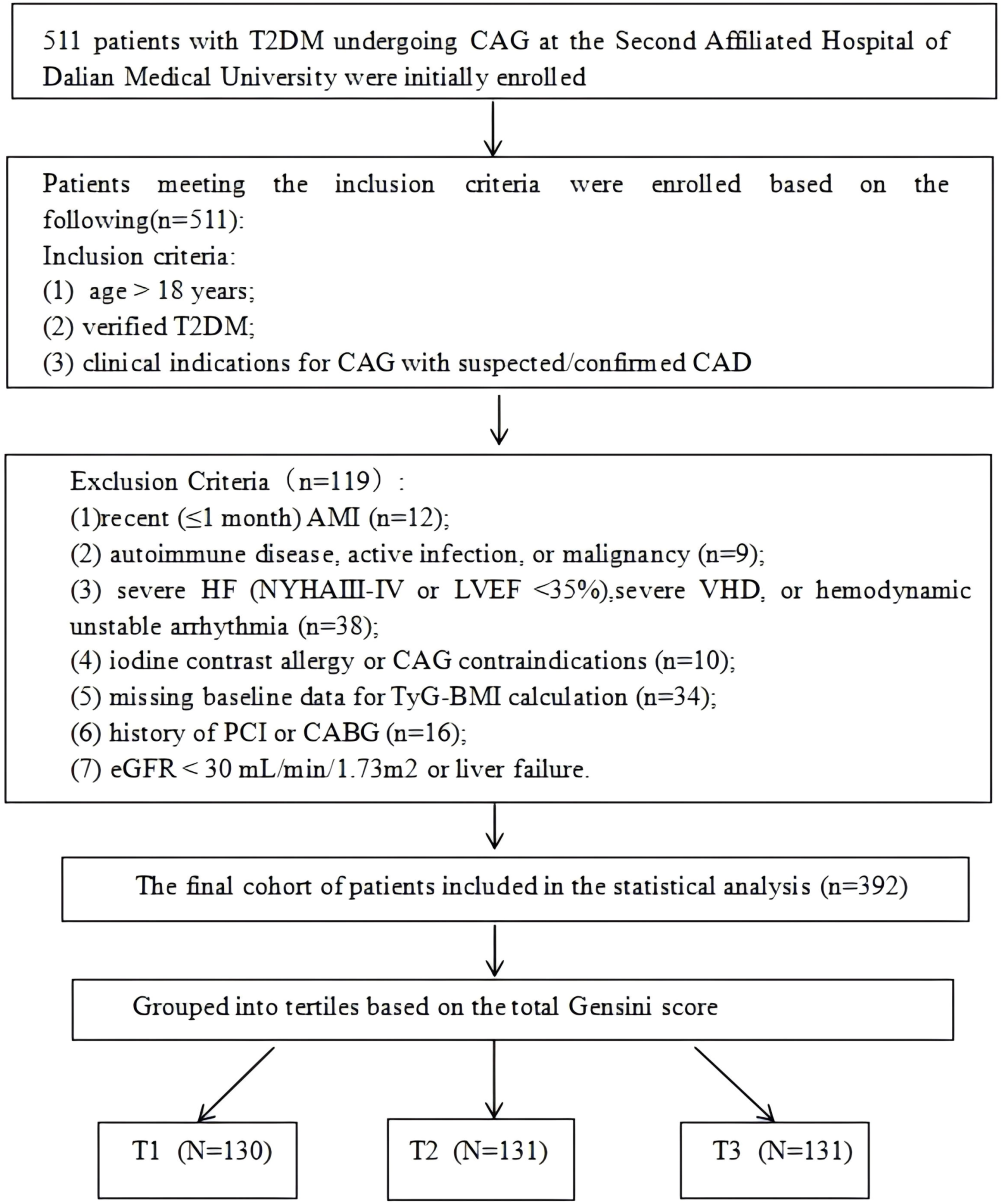
Flow diagram of participant selection.

This prospective, cohort study consecutively enrolled 511 patients with T2DM who underwent coronary angiography (CAG) at the Second Department of Cardiology, the Second Affiliated Hospital of Dalian Medical University, between November 1, 2025, and March 28, 2026. Consecutive sampling was employed to minimize selection bias. Eligible participants were adults aged >18 years with a confirmed diagnosis of type 2 diabetes mellitus (T2DM), established according to the American Diabetes Association diagnostic criteria, including a fasting plasma glucose ≥7.0 mmol/L, a 2-h plasma glucose ≥11.1 mmol/L during an oral glucose tolerance test, HbA1c ≥6.5%, or a documented history of T2DM receiving glucose-lowering therapy (14); clear clinical indications for CAG due to suspected or established coronary artery disease (15); and provision of written informed consent from patients or their legal representatives.The subsequent exclusion criteria were applied: recent acute myocardial infarction within 1 month (N = 12); concurrent autoimmune diseases, active infections, or malignancies (N = 9); severe heart failure (NYHA class III–IV or LVEF<35%), advanced valvular heart disease, or hemodynamically unstable arrhythmias (N = 38); documented iodine contrast allergy or CAG contraindications (N = 10); missing essential baseline data (TG, FBG, height, or weight) preventing TyG-BMI calculation (N = 34); and a history of PCI or CABG (N = 16). After strict exclusion according to the above criteria (some patients met multiple exclusion criteria simultaneously), a total of 392 patients were finally included for subsequent analysis. The detailed participant enrollment workflow is shown in **Figure 1**. This study strictly adhered to the ethical principles of the Declaration of Helsinki and received formal approval from the Ethics Committee of the Second Affiliated Hospital of Dalian Medical University (Approval No.: KY2025-648-01).

### 2.2 Data collection

Baseline characteristics and clinical data of the study population were comprehensively compiled, comprising: demographic profiles and medical histories (age, sex, smoking and alcohol consumption, and histories of hypertension and stroke); echocardiographic parameters [left ventricular ejection fraction (LVEF)], target vessel characteristics, and the severity of luminal stenosis; and antidiabetic medication regimens.All blood samples were analyzed at the Department of Clinical Laboratory, the Second Affiliated Hospital of Dalian Medical University, utilizing automated biochemical analyzers under standardized quality control protocols. The laboratory panel evaluated: complete blood counts (white blood cell, neutrophil, lymphocyte, and monocyte counts; red blood cell count, hemoglobin, and platelet count); and biochemical measurements, including fasting plasma glucose (FPG), glycated hemoglobin (HbA1c), triglycerides (TG), total cholesterol (TC), high-density lipoprotein cholesterol (HDL-C), low-density lipoprotein cholesterol (LDL-C), apolipoprotein A1 (ApoA1), apolipoprotein B (ApoB), uric acid (UA), and creatinine (Cr). All participants had a documented history of type 2 diabetes mellitus before enrollment. The diagnosis of T2DM was established according to recognized diagnostic criteria and confirmed through medical records. Importantly, most patients had been receiving long-term glucose-lowering treatment before admission, including insulin and oral hypoglycemic agents. Therefore, fasting plasma glucose and HbA1c values collected during hospitalization reflected glycaemic control under ongoing treatment rather than glycaemic status at the time of initial diagnosis.

### 2.3 Calculation of TyG-BMI index

To ensure consistency in concentration units across parameters, fasting plasma glucose (FPG) and triglycerides (TG) values were converted from mmol/L to mg/dL utilizing conversion factors of 18 and 88.57, respectively, as follows:FBG (mg/dL) = FBG (mmol/L)×18;TG (mg/dL) = TG (mmol/L)×88.57. The TyG-BMI index was subsequently calculated according to the established formula (16): TyG-BMI=Log[TG (mg/dL) ×FPG (mg/dL)/2]*BMI(kg/m^2^).

### 2.4 Serum collection and IQGAP3 quantification

For serum isolation, 5 mL of fasting venous blood was collected into anticoagulant-free procoagulation tubes, followed by a 30-minute incubation at room temperature and subsequent centrifugation at 3000 rpm for 15 minutes at 4 °C.

The resulting supernatant was aliquoted and preserved at -80 °C until further analysis. Serum IQGAP3 concentrations were quantified using a human IQGAP3 Enzyme-Linked Immunosorbent Assay (ELISA kit, Aimeng Youning, Shanghai, China) strictly according to the manufacturer’s protocol. Briefly, 50 μL of sample or standard was added to the reaction wells and incubated at 37 °C for 40 minutes. Following thorough washing, biotinylated antibodies and SABC working solution were sequentially applied with incubation periods of 30 and 20 minutes, respectively. After the final wash cycle, TMB substrate was added for color development in the dark, followed by the addition of a stop solution. Absorbance (optical density, OD) was measured at 450 nm. All assays were performed in duplicate to ensure experimental reproducibility.

### 2.5 Coronary severity and gensini score quantification

Coronary luminal stenosis was characterized based on the American College of Cardiology (ACC) standards, with its burden quantified via the Gensini score. The fundamental scoring rules were applied as follows: a luminal narrowing of<25% was allocated a score of 0; 25%–49%, a score of 1; 50%–74%, a score of 2; 75%–99%, a score of 3; and a 100% complete occlusion, a score of 4. Thereafter, adjusting for coronary anatomy, predetermined weighting coefficients were applied according to the specific lesion sites: a factor of 5 for left main trunk disease; 2.5 for proximal left anterior descending and circumflex artery lesions; 1.5 for mid-left anterior descending segments; and 1 for other arterial regions. The final Gensini score represents the aggregate value obtained from these eight individual target coronary segments (17).

To explore the association between serum IQGAP3, the TyG-BMI index, and coronary lesion severity, participants were further stratified into tertiles according to the distribution of Gensini scores within the study cohort. The cutoff values were defined as T1 (Gensini score ≤9), T2 (10–21), and T3 (>21). Of note, these tertile-based categories were derived from the internal distribution of the study population and therefore represent relative stratification of coronary lesion burden rather than externally validated or clinically established thresholds for severe coronary artery disease.

### 2.6 Statistical analysis

Statistical computing and graphics were executed using R Studio (version 4.5.2) and GraphPad Prism (version 10.1.2). Study participants were stratified into three groups based on Gensini score tertiles: the low-tertile group (T1, n=130), mid-tertile group (T2, n=131), and high-tertile group (T3, n=131). Normally distributed continuous variables are expressed as mean ± standard deviation ( $\bar{X}\pm \text{S}$) and compared using one-way ANOVA. Non-normally distributed data are presented as medians with interquartile ranges (IQRs), with intergroup differences analyzed via the Kruskal–Wallis H test. Categorical variables are summarized as frequencies and percentages [n (%)], using the chi-square test or Fisher’s exact test depending on expected cell counts.Inter-variable correlations were evaluated using Pearson correlation analysis and visualized via matrices heatmaps. To identify independent predictors, logistic regression analysis was performed with severe coronary atherosclerosis (T3 group vs. T1+T2 groups) as the binary outcome; variables yielding P< 0.05 in univariate analysis were entered into the multivariable model as covariates. Prior to multivariable modeling, collinearity diagnostics were performed, where a variance inflation factor (VIF)<5 indicated the absence of severe multicollinearity (**Table 1**). Restricted cubic splines (RCS) were modeled to examine and visualize potential non-linear relationships between serum IQGAP3, the TyG-BMI index, and the risk of severe coronary disease. Discriminative performance was appraised using the area under the receiver operating characteristic curve (AUC-ROC). Net reclassification index (NRI) and integrated discrimination improvement (IDI) were calculated to quantify the incremental diagnostic efficacy of the joint markers over the baseline risk model. To assess the robustness of the predictive model and minimize potential optimism arising from model development and evaluation within the same dataset, internal validation was performed using bootstrap resampling with 1,000 repetitions. Model discrimination was evaluated using the AUC, and optimism-corrected performance estimates were derived from bootstrap samples. Model calibration was assessed using a bootstrap-corrected calibration plot test.Finally, stratified subgroup analyses and interaction tests were performed across major clinical characteristics, with a forest plot demonstrating the consistency of effect sizes (ORs and 95% CIs). All statistical tests were two-tailed, and P< 0.05 was considered statistically significant.

**Table 1:** Collinearity analysis of variables.

Variable	VIF	Variable	VIF
Age	1.293	Cr	1.203
Gender	1.547	UA	1.238
Hypertension	1.082	Hb	2.048
Current Smoking	1.309	RBC	2.031
LVEF	1.168	WBC	1.125
TC	1.257	PLT	1.099
LDL-C	1.204	FBG	1.170
HDL-C	1.186	HbA1c	1.073
ALT	1.915	TyG-BMI	1.545
AST	1.870	IQGAP3	1.252

BMI, Body Mass Index; LVEF, left ventricular ejection fraction; TC, total cholesterol; LDL-C, low-density lipoprotein cholesterol; HDL-C, high-density lipoprotein cholesterol; Hb, hemoglobin; RBC, Red Blood Cell; WBC, White Blood Cell; PLT, platelet; UA, Uric Acid; ALT, alanineaminotransferase; AST, aspartateaminotransferase; Cr, creatinine; FPG, fasting plasma glucose.

## 3 Results

### 3.1 Baseline characteristics

**Figure 2:**
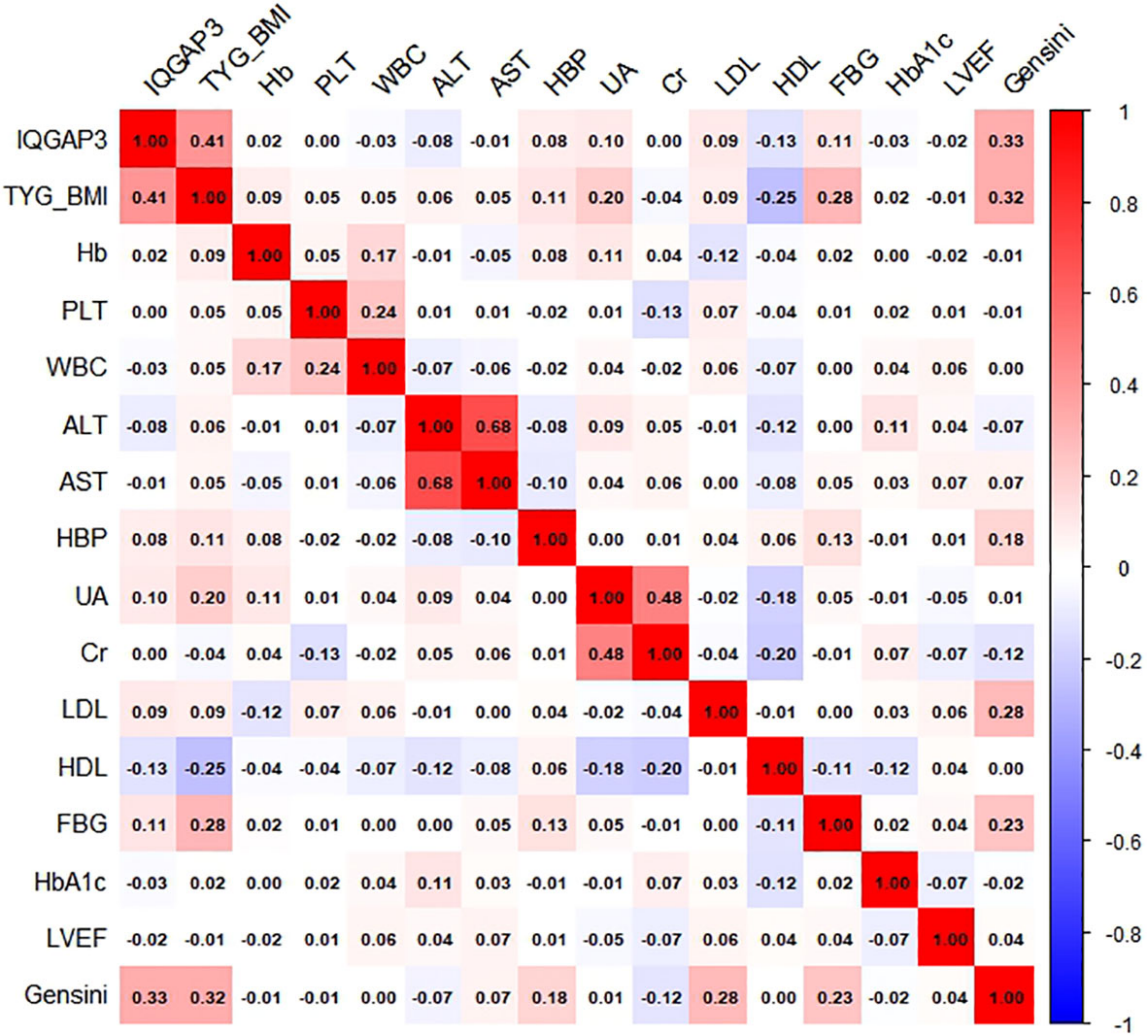
Correlation heatmap of variables.

A total of 392 patients with T2DM were enrolled in this study, presenting a male predominance of 60.2% and a mean age of 64.3 ± 11.7 years. Stratification by Gensini score tertiles partitioned the cohort into three groups: low (T1, n=130), mid (T2, n=131), and high (T3, n=131). As delineated in **Table 2**, statistically significant intergroup discrepancies were observed regarding age, BMI, and the prevalence of hypertension, alongside the levels of TG, LDL-C, FBG, the TyG-BMI index, and IQGAP3 (all P< 0.05). To further elucidate how serum IQGAP3 and the TyG-BMI index tie into the severity of coronary atherosclerosis, Pearson correlation analysis was executed to map the inter-variable associations (**Figure 2**). Regarding the core investigative markers, serum IQGAP3 exhibited a prominent positive correlation with the TyG-BMI index (r = 0.41). Notably, both IQGAP3 (r = 0.33) and the TyG-BMI index (r = 0.32) were coupled with the Gensini score in a clear, positive linear fashion (all P< 0.05), reinforcing that the up-regulated expression of these two indices tracks closely with advancing coronary artery disease burden. Parallel assessment of traditional cardiovascular risk factors revealed that the Gensini score also correlated positively with LDL-C (r = 0.28), FBG (r = 0.23), and hypertension (r = 0.18), while displaying a weak inverse relationship with Cr (r = - 0.12). Concurrently, the TyG-BMI index was tied to elevated FBG (r = 0.28) and UA (r = 0.20), but inversely linked with HDL-C (r = -0.25). Collectively, these bivariate insights confirm a robust linear alignment between serum IQGAP3, the TyG-BMI index, and coronary atherosclerotic severity, underscoring their clinical utility as potential circulatory biomarkers for staging coronary lesions in T2DM populations.

**Table 2:** Baseline characteristics.

Variable	All subjects (n=392)	T1 (n=130)	T2 (n=131)	T3 (n=131)	P-Value
Age,years	64.3 ± 11.7	62.3 ± 11.0	64.3 ± 11.7	66.4 ± 12.2	0.020
Man,n (%)	236(60.2)	79(60.8)	76(58.0)	81(61.8)	0.809
BMI	24.96 ± 2.83	23.87 ± 2.67	24.99 ± 2.71	26.00 ± 2.71	<0.001
Hypertension, n (%)	189(48.2)	50(38.5)	62(47.3)	77(58.8)	0.004
Current Smoking, n (%)	140(35.7)	40(30.8)	46(35.1)	54(41.2)	0.209
LVEF (%)	53.55 ± 6.67	53.82 ± 6.98	52.73 ± 6.72	54.11 ± 6.26	0.210
TC(mmol/L)	4.42 ± 1.24	4.36 ± 1.22	4.43 ± 1.23	4.48 ± 1.27	0.703
TG(mmol/L)	1.91 ± 1.49	1.68 ± 1.03	1.89 ± 0.99	2.15 ± 2.13	0.039
LDL-C(mmol/L)	2.68 ± 0.82	2.35 ± 0.70	2.66 ± 0.83	3.01 ± 0.81	<0.001
HDL-C(mmol/L)	0.99 ± 0.26	0.99 ± 0.24	0.99 ± 0.24	1.00 ± 0.30	0.935
Apo(a)(g/L)	1.18 ± 0.33	1.20 ± 0.35	1.17 ± 0.32	1.18 ± 0.33	0.786
Apo(b)(g/L)	0.97 ± 0.38	1.00 ± 0.40	0.97 ± 0.38	0.93 ± 0.35	0.271
ALT(U/L)	26.77 ± 14.75	28.27 ± 16.17	26.91 ± 14.52	25.13 ± 13.39	0.227
AST(U/L)	23.50 ± 10.02	23.01 ± 9.89	23.04 ± 10.03	24.26 ± 10.16	0.565
Cr (μmol/L)	77.03 ± 91.98	81.10 ± 88.79	78.75 ± 70.25	71.27 ± 52.66	0.516
UA(μmol/L)	339.46 ± 97.23	339.86 ± 106.78	345.36 ± 101.49	333.16 ± 97.23	0.597
Hb(g/L)	140.36 ± 16.36	141.92 ± 14.52	141.48 ± 17.05	139.84 ± 16.00	0.537
RBC(1012/L)	4.61 ± 0.62	4.67 ± 0.52	4.59 ± 0.73	4.56 ± 0.60	0.335
WBC(109/L)	6.66 ± 2.25	6.60 ± 2.26	6.87 ± 2.48	6.51 ± 1.99	0.404
PLT(109/L)	222.99 ± 57.16	229.86 ± 66.17	219.76 ± 55.21	219.39 ± 48.63	0.245
FBG (mmol/L)	5.84 ± 1.24	5.55 ± 1.33	5.82 ± 1.07	6.15 ± 1.25	<0.001
HbA1c(%)	5.30 ± 0.75	5.33 ± 0.74	5.29 ± 0.74	5.28 ± 0.78	0.821
Use of antidiabetic drugs					
Insulin,n (%)	181(46.2)	65(50.0)	55(42.0)	61(46.6)	0.428
Metformin,n (%)	299(76.3)	101(77.7)	100(76.3)	98(74.8)	0.861
SGLT-2i,n (%)	162(41.3)	53(40.8)	51(38.9)	58(44.3)	0.672
GLP-1 RA,n (%)	77(19.6)	24(18.5)	23(17.6)	30(22.9)	0.507
Other,n (%)	181(46.2)	58(44.6)	67(51.1)	56(42.7)	0.359
TyG-BMI	222.09 ± 31.55	208.91 ± 31.00	223.44 ± 26.99	233.81 ± 31.57	<0.001
IQGAP3(ng/ml)	1.38 ± 0.69	1.12 ± 0.57	1.28 ± 0.68	1.74 ± 0.65	<0.001
Gensini score	20.23 ± 18.13	6.18 ± 1.96	14.47 ± 3.60	39.94 ± 18.71	<0.001

P values< 0.05. Patients were stratified into tertiles according to the Gensini score: T1, T2, and T3 groups. BMI, Body Mass Index; LVEF, left ventricular ejection fraction; TC, total cholesterol; TG, triacylglycerol; LDL-C, low-density lipoprotein cholesterol; HDL-C, high-density lipoprotein cholesterol; Hb, hemoglobin; WBC, White Blood Cell; PLT, platelet; AST, aspartateaminotransferase; Cr, creatinine; ALT, alanineaminotransferase; FPG, fasting plasma glucose.

### 3.2 Multivariate logistic regression analysis

After adjusting for potential confounding variables, the multivariable logistic regression analysis (**Table 3**) identified several independent predictors tracking with the severity of coronary artery disease. Notably, older age (OR = 1.037, 95% CI: 1.014–1.061, P = 0.002), a history of hypertension (OR = 1.673, 95% CI: 1.021–2.741, P = 0.041), elevated LDL-C levels (OR = 2.078, 95% CI: 1.528–2.824, P< 0.001), a higher TyG-BMI index (OR = 1.012, 95% CI: 1.003–1.021, P = 0.006), and up-regulated IQGAP3 expression (OR = 3.119, 95% CI: 2.049–4.747, P< 0.001) remained robustly linked to advanced lesions.Quantitatively, each incremental year of age exhibited a 3.7% higher risk of severe coronary stenosis. Hypertensive status translated to a 1.673-fold higher likelihood of presenting severe lesions relative to normotensive counterparts. Each 1-unit increase in the TyG-BMI index was associated with a 1.2% increase in risk. Similarly, each 1-unit increase in serum IQGAP3 (ng/mL) was associated with approximately a three-fold increase (OR = 3.119) in the odds of severe coronary lesions. Conversely, male sex (OR = 1.073, 95% CI: 0.607–1.895, P = 0.809) and smoking history (OR = 1.095, 95% CI: 0.625–1.915, P = 0.752) yielded no statistically significant correlation with disease severity.

**Table 3:** Univariate and multivariate logistic regression analysis.

Variable	Univariate analysis		Multivariate analysis	
	OR (95% CI)	P value	OR (95% CI)	P value
Age	1.024(1.005-1.043)	0.015	1.037(1.014-1.061)	0.002
Man	1.108(0.720-1.704)	0.641	1.073(0.607-1.895)	0.809
Hypertension	1.897(1.239-2.903)	0.003	1.673(1.021-2.741)	0.041
Current Smoking	1.427(0.925-2.201)	0.108	1.095(0.625-1.915)	0.752
LVEF	1.020(0.987-1.053)	0.241		
TC	1.063(0.897-1.259)	0.482		
LDL-C	2.179(1.645-2.886)	<0.001	2.078(1.528-2.824)	<0.001
HDL-C	1.162(0.521-2.591)	0.714		
ALT	0.988(0.974-1.003)	0.121		
AST	1.011(0.991-1.032)	0.289		
Cr	0.998(0.993-1.002)	0.295		
UA	0.999(0.997-1.001)	0.363		
Hb	0.993(0.98-1.006)	0.274		
RBC	0.841(0.601-1.176)	0.311		
WBC	0.955(0.866-1.052)	0.351		
PLT	0.998(0.995-1.002)	0.377		
HbA1c	0.937(0.709-1.240)	0.651		
TyG-BMI	1.018(1.011-1.026)	<0.001	1.012(1.003-1.021)	0.006
IQGAP3	3.605(2.490-5.221)	<0.001	3.119(2.049-4.747)	<0.001

P values< 0.05. BMI, Body Mass Index; LVEF, left ventricular ejection fraction; TC, total cholesterol; LDL-C, low-density lipoprotein cholesterol; HDL-C, high-density lipoprotein cholesterol; Hb, hemoglobin; WBC, White Blood Cell; PLT, platelet; AST, aspartateaminotransferase; Cr, creatinine; ALT, alanineaminotransferase.

### 3.3 Incremental predictive value of TyG-BMI and IQGAP3 beyond conventional risks

**Figure 3:**
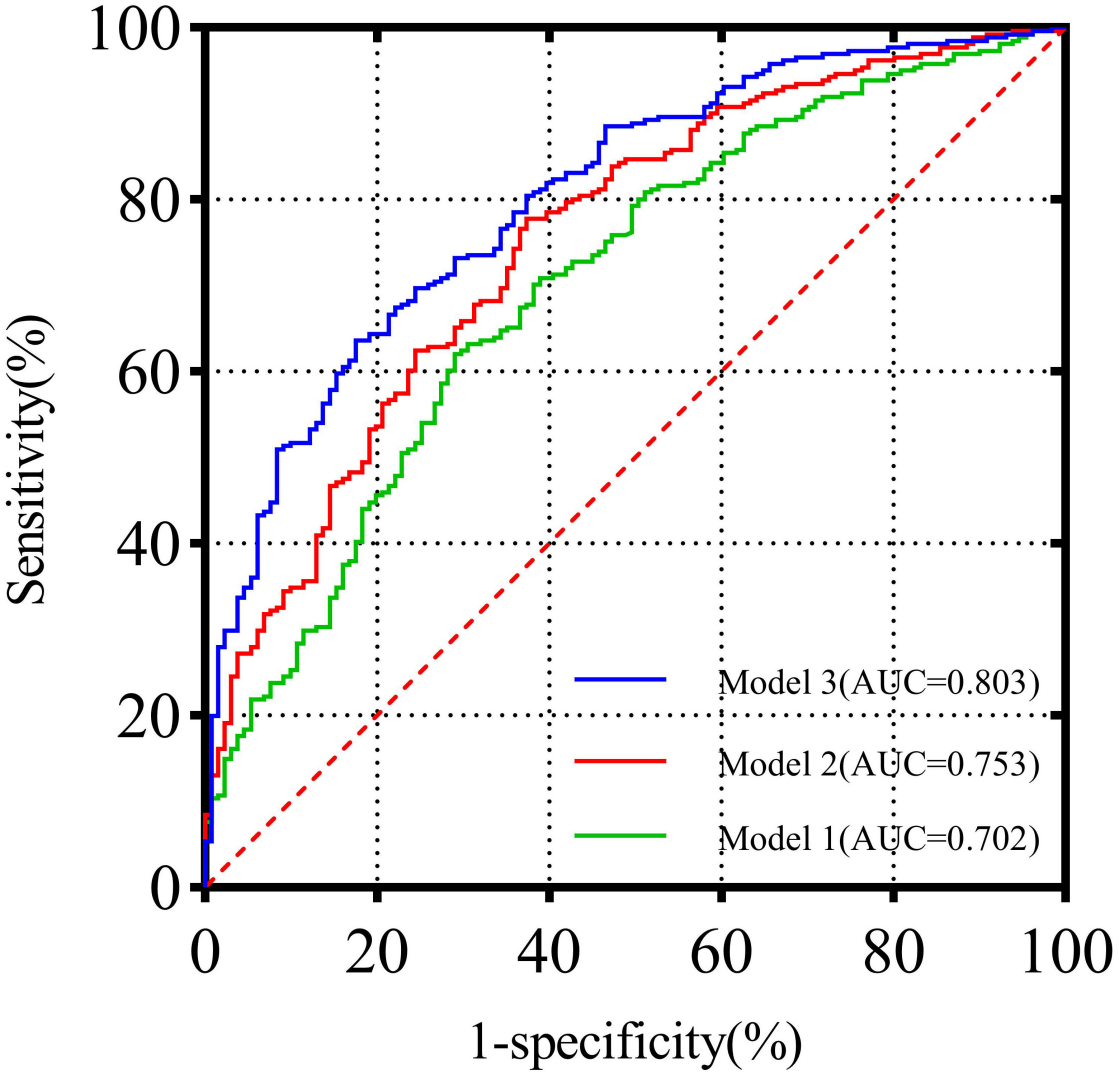
ROC curve analysis of the three models.

To evaluate the incremental predictive value of the TyG-BMI index and serum IQGAP3 levels over established indicators for coronary severity, three progressive logistic regression models were constructed. Based on clinical consensus, male sex and current smoking status were retained within the baseline model as traditional risk factors to rigorously minimize potential confounding bias. The comparative discriminative and reclassification performance across these models is displayed via the ROC curves (**Figure 3**) and summarized in **Table 4**.The baseline model (Model 1), which adjusted for conventional risk factors—including male sex, age, history of hypertension, current smoking, and LDL-C—demonstrated moderate discriminative capacity, yielding an area under the curve (AUC) of 0.702 (95% CI: 0.648–0.757, P< 0.001). The integration of the TyG-BMI index into Model 1 (yielding Model 2) bolstered the discriminative power, as evidenced by an expanded AUC of 0.753 (95% CI: 0.702–0.803, P< 0.001). Compared with Model 1, Model 2 exhibited superior reclassification efficacy, with a net reclassification index (NRI) of 0.180 (95% CI: 0.094–0.267, P< 0.001) and an integrated discrimination improvement (IDI) of 0.065 (95% CI: 0.039–0.091, P< 0.001). Crucially, layering the novel molecular marker IQGAP3 onto Model 2 (Model 3) achieved the peak predictive performance, where the AUC augmented further to 0.803 (95% CI: 0.758–0.847, P< 0.001). Relative to Model 2, which relied solely on clinical and metabolic parameters, Model 3 elicited a more pronounced optimization in both reclassification and discrimination metrics, showing an NRI of 0.307 (95% CI: 0.201–0.407, P< 0.001) and an IDI of 0.138 (95% CI: 0.101–0.174, P< 0.001). These findings indicate that cascading a traditional metabolic index (TyG-BMI) and a peripheral molecular biomarker (IQGAP3) onto conventional cardiovascular vectors markedly refines clinical screening and staging capabilities for coronary atherosclerosis burden.

**Table 4:** Evaluation of predictive models.

Variables	NRI		IDI		C-statistics	
	Index(95%CI)	P value	Index(95%CI)	P value	Index(95%CI)	P value
Model 1		ref		ref	0.702 (0.648-0.757)	<0.001
Model 2	0.180 (0.094-0.267)	<0.001	0.065 (0.039-0.091)	<0.001	0.753 (0.702-0.803)	<0.001
Model 3	0.307 (0.201-0.407)	<0.001	0.138 (0.101-0.174)	<0.001	0.803 (0.758-0.847)	<0.001

Model 1: Gender + age + Hypertension + Current smoking + LDL-C.

Model 2: Gender + age + Hypertension + Current smoking + LDL-C + TyG-BMI.

Model 3: Gender + age + Hypertension + Current smoking + LDL-C + TyG-BMI + IQGAP3.

**Figure 4:**
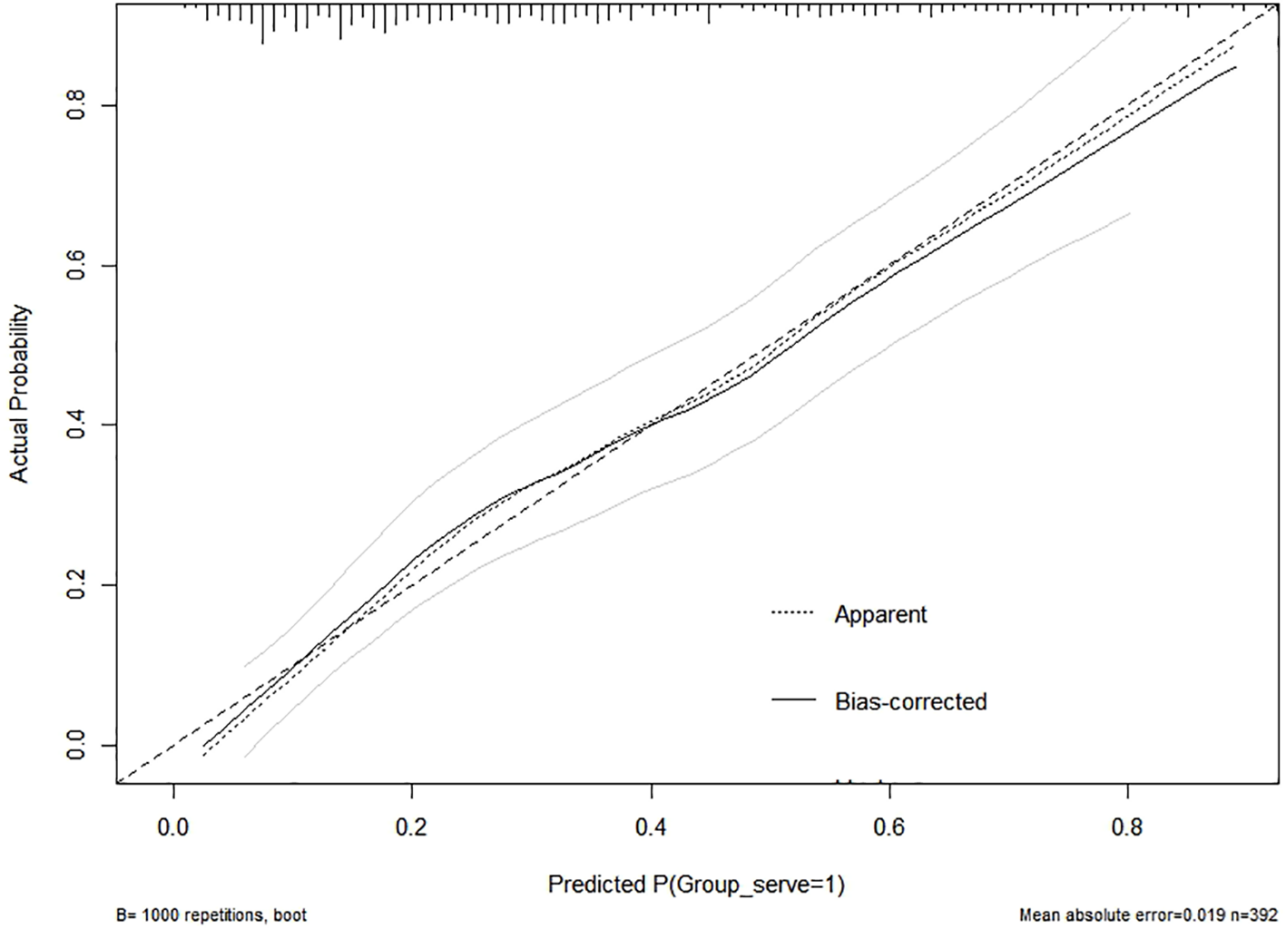
Calibration plot of the combined model.

To further evaluate the robustness of the combined model, bootstrap internal validation with 1,000 resamples was performed. The apparent AUC was 0.803, and the optimism-corrected AUC remained 0.788, indicating minimal overfitting and satisfactory model stability. The bootstrap-corrected calibration slope was 0.926. Calibration analysis demonstrated good agreement between predicted and observed probabilities (**Figure 4**). The mean absolute error and mean squared error were 0.019 and 0.00046, respectively, while the 90th percentile of absolute prediction error was 0.033. The calibration plot further confirmed adequate calibration of the model.

### 3.4 Dose-response trajectories via restricted cubic spline analysis for coronary severity in T2DM patients

**Figure 5:**
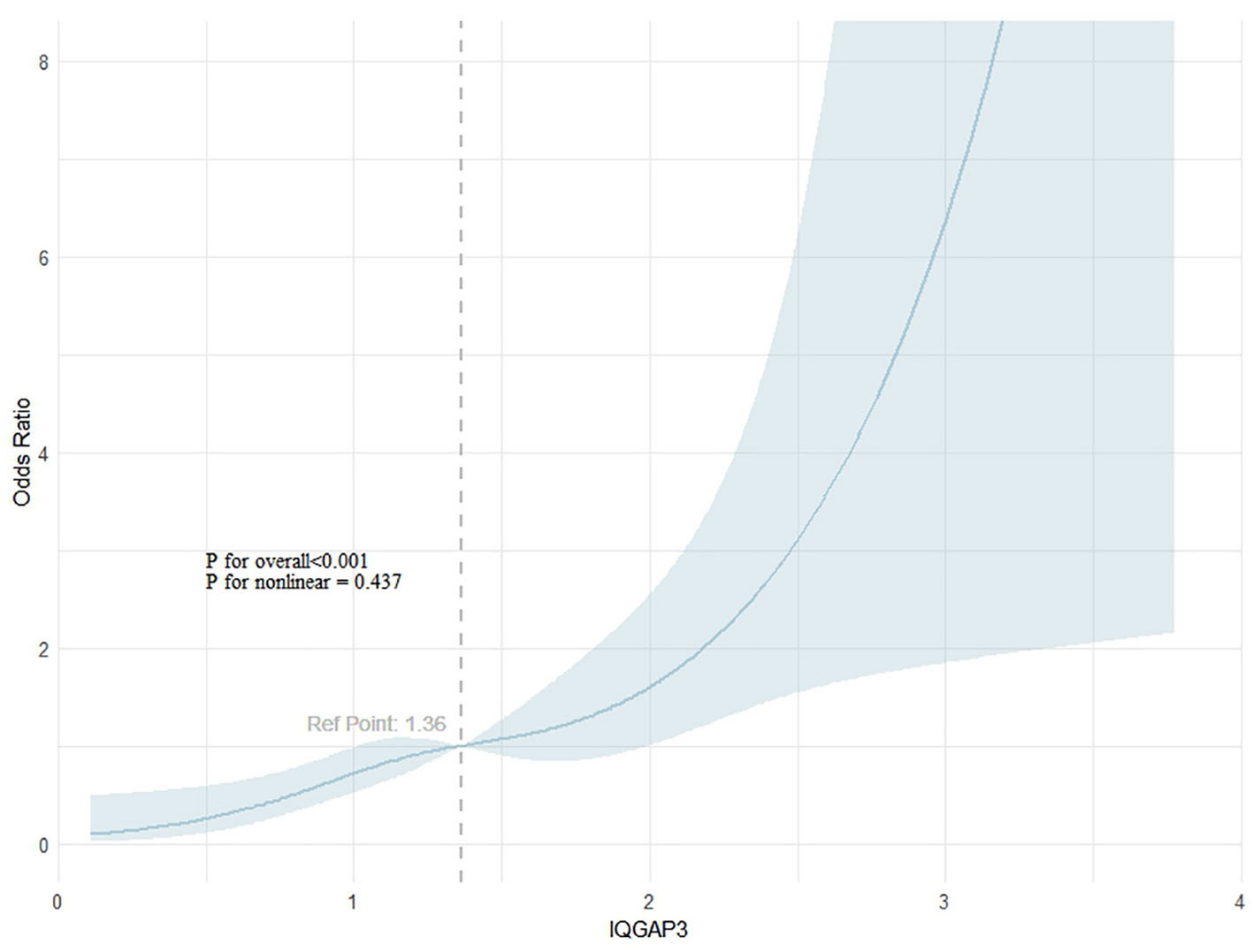
RCS of IGGAP3 and CHigh gensini score (T3).

**Figure 6:**
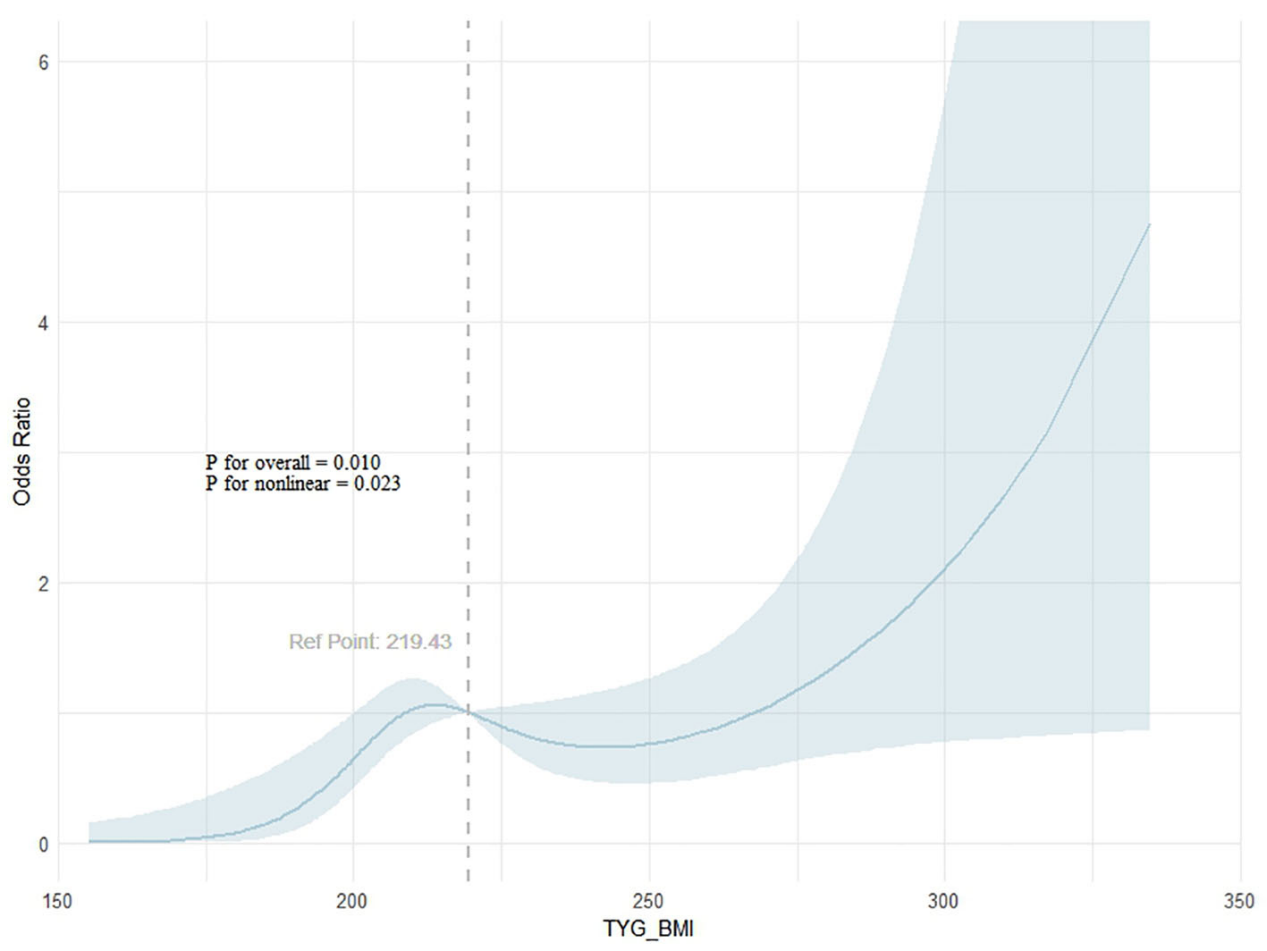
RCS of TyG-BMI and high gensini score (T3).

To elucidate the potential dose-response profiles, restricted cubic splines (RCS) were configured to model the risk trajectories (expressed as odds ratios) of serum IQGAP3 levels and the TyG-BMI index relative to coronary atherosclerosis severity in patients with T2DM (**Figures 5**, **6**). The spline configurations unveiled a prominent linear association between serum IQGAP3 and disease severity (P_overall_< 0.001, P_nonlinear_ = 0.437). Along with the up-regulated expression of IQGAP3, the probability of presenting severe coronary atherosclerosis exhibited a steady and unremitting, quasi-linear escalation. Conversely, the TyG-BMI index was bound to severe coronary disease risk via a definitive non-linear dose-response trajectory punctuated by a threshold effect (P_overall_ = 0.010, P_nonlinear_= 0.023). Specifically, after crossing this clinical inflection point into the upper-tier range, the propensity for aggravated coronary lesions tracked an upward velocity. These RCS-derived patterns reinforce that high levels of both markers are independently tied to advanced coronary pathology. Their divergent graphical modalities—characterized by persistent linear progression versus non-linear threshold evolution—reflect complementary pathophysiological dimensions, statistically validating the clinical utility of their combined deployment for the synergistic staging of coronary atherosclerosis burden in T2DM.

### 3.5 Subgroup stratification and cross-over interaction diagnostics

**Figure 7:**
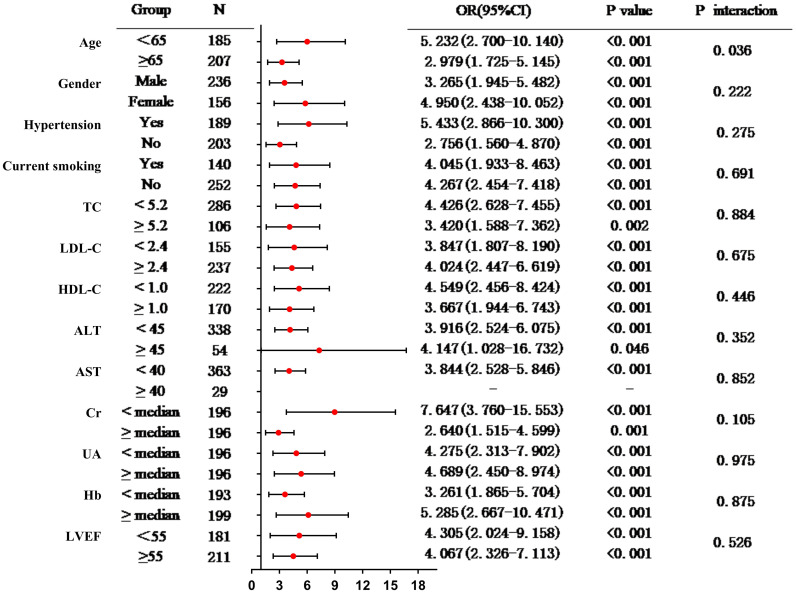
Subgroup analyses for IQGAP3 and high gensini score (T3).

**Figure 8:**
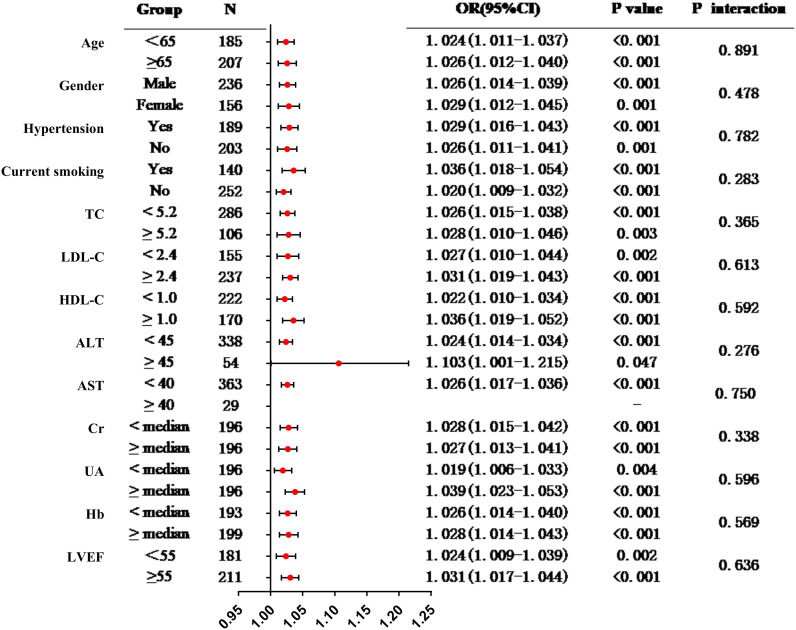
Subgroup analyses for TyG-BMI and high gensini Score (T3).

To substantiate the analytical robustness of serum IQGAP3 and the TyG-BMI index in staging coronary severity among T2DM patients, stratified subgroup analyses were executed across major clinical phenotypes, including age, sex, hypertensive status, smoking cohorts, lipid portfolios, hepato-renal indices, and left ventricular ejection fraction (**Figures 7**, **8**). The independent positive correlation between elevated serum IQGAP3 and severe coronary risk remained highly resilient across the vast majority of analyzed strata (OR>1, P<0.05). Notably, interaction diagnostics revealed that chronological age acted as a significant effect modifier on the predictive capacity of IQGAP3 (P_interaction_= 0.036). The risk effect of IQGAP3 was remarkably pronounced among younger counterparts aged< 65years (OR = 5.232, 95% CI: 2.700-10.140, P< 0.001); in patients aged >65 years, this association attenuated but maintained full statistical significance (OR = 2.979, 95% CI: 1.725-5.145, P< 0.001). Beyond age, no significant cross-over interactions were detected between any clinical covariates and serum IQGAP3 (all P_interaction_> 0.05). Parallel resilience was documented for the TyG-BMI index, which maintained a widespread association with heightened coronary risks (OR > 1, P< 0.05). Further interaction testing demonstrated that age, gender, comorbidities, and biochemical substrates exerted no notable modification on the performance of the TyG-BMI index (all P_interaction_> 0.05), highlighting that its predictive fidelity remains highly uniform across heterogeneous clinical backgrounds. Collectively, these subgroup and interaction configurations establish that both serum IQGAP3 and the TyG-BMI index possess exceptional clinical applicability and predictive stability as circulatory gauges for monitoring coronary atherosclerotic staging in T2DM.

## 4 Discussion

To our knowledge, this study is the first to substantiate that the combination of serum IQGAP3 and the TyG-BMI index acts as an independent predictor of coronary artery disease severity in patients with T2DM. This joint predictive fidelity was robustly sustained even after rigorous adjustments for HbA1c—reflecting chronic glycemic excursions—and glucose-lowering therapeutic regimens, underscoring the cumulative metabolic-vascular insult driving coronary pathology. Notably, the absence of significant differences in HbA1c among Gensini tertiles suggests that the association between TyG-BMI, IQGAP3 and coronary lesion severity cannot be simply explained by conventional glycaemic control, supporting their potential value as markers beyond HbA1c. RCS analysis further unveiled an asymmetric complementarity between these two parameters: serum IQGAP3 exhibited a stable, positive linear alignment with coronary stenosis severity, whereas the TyG-BMI index demonstrated an exponential risk surge after crossing a distinct clinical threshold. These divergent mathematical trajectories provide a robust statistical foundation for their synergistic diagnostic efficacy.Notably, a prominent interaction crossed between chronological age and IQGAP3, whereby the predictive effect was substantially pronounced in younger counterparts aged< 65 years. This age-specific divergence implies that in older populations, the cumulative burden of long-term vascular aging, calcification, and traditional risk vectors over decades dilutes the distinct molecular signature of IQGAP3. Conversely, in the younger cohort, IQGAP3-mediated vascular remodeling appears to represent an active, primary pathophysiological driver of atherogenesis, highlighting its paramount clinical utility for early risk stratification in this vulnerable subset.

Accelerated progress of atherosclerosis in patients with T2DM is often predominantly driven by the microenvironmental deterioration secondary to chronic hyperglycemia and hyperinsulinemia (18). Specifically, sustained hyperglycemia not only tends to exacerbate the vascular release of ROS and inflammatory infiltration, but may also unfavorably alter lipid profiles, characterized by elevated proportions of total cholesterol, triglycerides, and chylomicrons. These interlinked metabolic insults are thought to compromise endothelial structural and functional integrity, potentially accelerating plaque formation while concomitantly triggering proliferative cascades within smooth muscle cells—collectively underpinning a central nexus of diabetic vasculopathy (19, 20). Within the context of metabolic derangements and microenvironmental evolution, the identification of reliable cardiovascular biomarkers has garnered substantial interest. Although IQGAP3 is more frequently documented in oncological literature, its involvement in cardiovascular remodeling and metabolic inflammation is gradually becoming apparent (21, 22). Recent evidence indicates that Wilms tumor 1-associating protein (WTAP) modulates the dynamic equilibrium of endothelial stress fibers via the KLF2/IQGAP3 axis, implying a close involvement in the initiation and progression of cardiovascular disease (23). Concurrently, injury to vascular endothelial cells (VECs) and VSMCs provoked by high-glucose conditions is widely recognized as a pivotal component of AS pathology (24), with IQGAP3 serving as a candidate molecular mediator of cellular phenotypic switching and migration within this altered microenvironment (25). In light of the statistical insights from the present study, serum IQGAP3 correlates closely with the extent of coronary stenosis. This observation raises the possibility that as coronary atherosclerotic lesions advance, and local myocardial tissues adapt to an inflammatory and hypoxic microenvironment, the vascular wall might release IQGAP3 either as a compensatory response or as a spillover from tissue injury. Consequently, fluctuations in peripheral serum IQGAP3 levels could potentially mirror the pathological severity of plaque fibrous cap evolution, though this tentative mechanism warrants robust validation in prospective investigations.

As a novel surrogate marker for evaluating insulin resistance (IR), the TyG-BMI index has recently gained substantial recognition regarding its potential clinical utility in predicting adverse cardiovascular events (26, 27). Compared to the traditional homeostasis model assessment of insulin resistance (HOMA-IR), this index integrates fasting plasma glucose and peripheral triglycerides alongside the physiological impact of adiposity. Consequently, it may offer a more comprehensive reflection of the underlying IR state—a core pathological hallmark of T2DM that is thought to chronically drive the progression of CAD (28). Prior investigations have indicated that patients with multivessel disease tend to exhibit significantly elevated TyG-BMI levels relative to those with single-vessel lesions, suggesting that an abnormal elevation of this index is likely tied to an independent risk increment for multivessel coronary involvement (16). The findings of the present study appear to further contextualize and extend these conclusions, identifying a positive alignment of the TyG-BMI index, when combined with IQGAP3, with the severity of coronary stenosis in a diabetic population. From a pathophysiological perspective, worsening peripheral insulin sensitivity is highly likely to initiate a cascade of hepatic steatosis and systemic metabolic derangements. This process potentially accelerates atherosclerotic plaque evolution via lipotoxicity cascades and chronic inflammation-mediated endothelial injury (29).

The rationale for combining serum IQGAP3 with the TyG-BMI index in this study lies in the complementary insights offered by their distinct monitoring dimensions. The TyG-BMI index predominantly captures systemic metabolic aberrations derived from long-term insulin resistance and obesity (30), yet it may lack the fidelity to directly mirror localized vascular wall proliferation and plaque dynamics. Conversely, while IQGAP3 is thought to reflect localized vascular remodeling (31), it does not account for the broader systemic lipid and glucose metabolic background. The integration of these two parameters effectively bridges the metabolic “ etiology” with the structural “outcomes” within the vascular wall. This concept bolsters the insights gained from our ROC curve analysis, where the combined predictor demonstrated an expanded AUC for differentiating mild from severe coronary stenosis compared to either parameter alone, further supporting the presence of a complementary and synergistic efficacy brought by this dual-panel approach.

## 5 Limitations

Although this prospective study demonstrates the clinical value of combined serum IQGAP3 and TyG-BMI index in assessing coronary plaque burden and lesion severity in patients with T2DM, several limitations warrant consideration. First, the inherent constraints of a single-center design may introduce selection bias, as the cohort derived from a single institution possesses substantial homogeneity in dietary habits, lifestyles, and regional characteristics. Given that TyG-BMI—a composite metabolic parameter—is highly susceptible to environmental variations, the generalizability of our findings requires multi-center validation. Second, the modest sample size restricted aggressive subgroup stratifications. Consequently, we could not perform dedicated analyses on high-risk, complex anatomical phenotypes such as left main disease, diffuse calcification, or bifurcation lesions, leaving the refined predictive efficacy of the joint model in these critical subgroups uncharacterized.

From a methodological standpoint, the static, cross-sectional measurement of serum IQGAP3 and TyG-BMI at baseline constitutes a key vulnerability. Because both markers fluctuate over time, our reliance on a single admission sample failed to capture longitudinal trajectories under the influence of subsequent medical therapies (e.g., intensive glucose- or lipid-lowering drugs) or revascularization. Thus, the dynamic correlation between biomarker evolution and vascular remodeling remains unexplored. Finally, this study lacks longitudinal follow-up for hard clinical endpoints, relying instead on the Gensini score as a surrogate angiographic milestone. The brief observation window precluded the direct evaluation of the long-term causal relationship between this joint model and the future incidence of major adverse cardiovascular and cerebrovascular events (MACCE). Future large-scale, multi-center prospective trials utilizing serial biomarker tracking and long-term event-driven outcomes are required to fully establish the clinical utility of this dual-parameter approach.

## 6 Conclusion

Our findings suggest that serum IQGAP3 levels and the TyG-BMI index exhibit a mutually complementary relationship in evaluating coronary atherosclerotic burden and structural remodeling among patients with type 2 diabetes mellitus (T2DM). By integrating a localized biomarker of vascular wall remodeling with a composite index of systemic metabolic dysregulation, this joint approach offers a pragmatic framework to bridge local plaque characteristics and systemic metabolic states. Clinically, the combined model demonstrates improved discriminative performance for coronary lesion severity compared with either parameter alone. Consequently, it holds promising potential as a convenient, non-invasive tool to support early risk stratification and risk recognition in high-risk T2DM cohorts with suspected coronary artery disease.

## Data Availability

The original contributions presented in the study are included in the article/supplementary material. Further inquiries can be directed to the corresponding author.
